# Occurrence of Antibiotic Resistance Genes in *Hermetia illucens* Larvae Fed Coffee Silverskin Enriched with *Schizochytrium limacinum* or *Isochrysis galbana* Microalgae

**DOI:** 10.3390/genes12020213

**Published:** 2021-02-01

**Authors:** Vesna Milanović, Andrea Roncolini, Federica Cardinali, Cristiana Garofalo, Lucia Aquilanti, Paola Riolo, Sara Ruschioni, Lorenzo Corsi, Nunzio Isidoro, Matteo Zarantoniello, Ike Olivotto, Simone Ceccobelli, Stefano Tavoletti, Francesca Clementi, Andrea Osimani

**Affiliations:** 1 Department of Agricultural, Food and Environmental Sciences, Polytechnic University of Marche, via Brecce Bianche, 60131 Ancona, Italy; v.milanovic@staff.univpm.it (V.M.); a.roncolini@pm.univpm.it (A.R.); f.cardinali@staff.univpm.it (F.C.); c.garofalo@staff.univpm.it (C.G.); l.aquilanti@staff.univpm.it (L.A.); p.riolo@staff.univpm.it (P.R.); s.ruschioni@staff.univpm.it (S.R.); lorenzo.corsi1993@gmail.com (L.C.); n.isidoro@staff.univpm.it (N.I.); s.ceccobelli@staff.univpm.it (S.C.); s.tavoletti@staff.univpm.it (S.T.); f.clementi@staff.univpm.it (F.C.); 2 Department of Life and Environmental Sciences, Polytechnic University of Marche, via Brecce Bianche, 60131 Ancona, Italy; m.zarantoniello@pm.univpm.it (M.Z.); i.olivotto@staff.univpm.it (I.O.)

**Keywords:** *Hermetia illucens*, antibiotic resistance genes, rearing substrates, microalgae, coffee silverskin, frass

## Abstract

*Hermetia illucens* larvae are among the most promising insects for use as food or feed ingredients due to their ability to convert organic waste into biomass with high-quality proteins. In this novel food or feed source, the absence of antibiotic-resistant bacteria and their antibiotic resistance (AR) genes, which could be horizontally transferred to animal or human pathogens through the food chain, must be guaranteed. This study was conducted to enhance the extremely scarce knowledge on the occurrence of AR genes conferring resistance to the main classes of antibiotics in a rearing chain of *H. illucens* larvae and how they were affected by rearing substrates based on coffee silverskin supplemented with increasing percentages of *Schizochytrium limacinum* or *Isochrysis galbana* microalgae. Overall, the PCR and nested PCR assays showed a high prevalence of tetracycline resistance genes. No significant effect of rearing substrates on the distribution of the AR genes in the *H. illucens* larvae was observed. In contrast, the frass samples were characterized by a significant accumulation of AR genes, and this phenomenon was particularly evident for the samples collected after rearing *H. illucens* larvae on substrates supplemented with high percentages (>20%) of *I. galbana.* The latter finding indicates potential safety concerns in reusing frass in agriculture.

## 1. Introduction

The global population is estimated to reach 9 billion by 2050; hence, increasing amounts of animal feed and human food will be necessary to meet the demand of the growing population [1]. It is noteworthy that the production of animal feed is increasing the pressure on the environment by competing for water, land, and energy resources with human food production, both generating significant quantities of byproducts [1]. In such a context, edible insects can be used for the conversion of organic byproducts into valuable insect biomass rich in protein and fat that can be further used for human and animal nutrition [2,3]. Moreover, the production of insects is characterized by a low environmental impact in terms of feed conversion efficiency, a limited need for water and land sources and low CO_2_ and greenhouse gas emissions [4]. These characteristics are particularly important for aquaculture, where scarce and expensive fishmeal needs to be replaced with alternative high-protein feed to respond to the increasing world demand for fish [4,5].

In the European Union (EU), insect proteins from black soldier flies (*Hermetia illucens*), common houseflies (*Musca domestica*), lesser mealworms (*Alphitobius diaperinus*), yellow mealworms (*Tenebrio molitor*), banded crickets (*Gryllodes sigillatus*), field crickets (*Gryllus assimilis*), and house crickets (*Acheta domesticus*) have been authorized for use in aquaculture by the European Commission Regulation (EU) 2017/893 since mid-2017 [6]. Among these, *H. illucens* larvae are among the most promising insects to be used as feed, principally due to their good capability of converting a wide variety of organic wastes, such as coffee, vegetables, fruit, fish, urban organic wastes, and animal manure into the organic matter of the larval body, which is mainly composed of valuable proteins (approximately 55% of body dry weight (BDW)) and lipids (approximately 35% BDW) [7]. The nutritional quality of *H. illucens* biomass is strongly influenced by the diet [8]. Despite high lipid content of the larvae, their fatty acid composition is characterized by low amounts of polyunsaturated (PUFA) and monounsaturated (MUFA) fatty acids. In this regard, Truzzi et al. [9] demonstrated that the substrates enriched with marine microalgae such as *Schizochytrium* sp. or *Isochrysis* sp. can significantly increase the relative quantity of lipids and proteins in *H. illucens* larvae, thus improving their nutritional value. Moreover, *H. illucens* has been reported to be able to degrade mycotoxins, pesticides, and tetracyclines as well as to remove heavy metals from contaminated wastes [10,11].

Living organisms including insects are natural carriers of microorganisms. The species-specific microbiota of insects may be altered by different extrinsic factors, such as diet, rearing conditions, handling, and processing [12]. Bacteria linked to edible insects could also be a reservoir of antibiotic resistance (AR) genes conferring resistance to antibiotics commonly used in human and veterinary medicine, agriculture, and aquaculture, whose extensive use has triggered selective pressure leading to the development of antibiotic-resistant strains [12]. Different public health organizations, such as the World Health Organization (WHO), the European Food Safety Authority (EFSA), the European Center for Disease Prevention and Control (ECDC) and the European Medicines Agency (EMA), have identified antibiotic resistance as a global threat to public health, giving special attention to the spread of AR genes via horizontal gene transfer among bacteria from the same or different species [13].

Previous studies were mainly focused on the presence of AR genes in small cricket powder, locusts, black ants, giant water bugs, rhino beetles, termite alates, silkworm pupae, mole crickets and black scorpions [14]; grasshoppers [15,16]; mealworms [14,15,17,18]; and small crickets [14,18,19], all reporting a high prevalence of genes conferring resistance to tetracyclines and erythromycin. The possible contribution of feed to the occurrence of AR genes in edible insects as well as their vertical transmission via insect egg smearing has already been reported by Osimani et al. [20]. To the best of the author’s knowledge, very few studies have been conducted on the occurrence of AR genes in *H. illucens* larvae [10,21] and even fewer have been conducted on the effect of rearing substrate on the distribution of AR genes in the larvae as well as their possible accumulation in the frass (a mixture of insect excrements and substrate residues) [22]. It is noteworthy that two conversion cycles are needed when insects are used as food or feed, specifically, the conversion of organic products into insect biomass and then from insects to animals or humans [23]; hence, constant monitoring of the possible sources of AR genes to plan interventions aimed at reducing the spread of AR genes among feed and food chains should be a priority [13].

Given the above premises, the present study was designed to elucidate the possible effect of rearing substrates on the occurrence of selected AR genes in *H. illucens* larvae as well as on their accumulation or even depletion in frass. In the concept of circular economy, the *H. illucens* larvae were reared on a basal substrate constituted by coffee roasting byproducts (coffee silverskin, Cs) supplemented with increasing percentages of microalgae (*Schizochytrium limacinum* or *Isochrysis galbana*) as the source of PUFAs such as docosahexaenoic, stearidonic, and α-linolenic acid [9,24]. On the other hand, Cs, rich in polyphenols and mainly composed of dietary fibers (≈55%), proteins (≈19%), carbohydrates (≈6%), and fat (≈2%), has been reported to have prebiotic and antioxidant activities [25].

The DNA extracted from the rearing substrates, larvae and frass samples was screened for the presence of 12 AR genes conferring resistance to five key classes of antibiotics commonly used in human and veterinary medicine as well as in agriculture and aquaculture, such as erythromycin (*erm*(A), *erm*(B), *erm*(C)), vancomycin (*vanA*, *vanB*), tetracycline [*tet*(K), *tet*(M), *tet*(O), *tet*(S)], β-lactams (*blaZ, mecA*), and aminoglycosides (*aac*(6′)-*le* *aph*(2″)-*la*, referred to as *aac-aph*). The target AR genes were selected from among those frequently found in bacteria isolated from various animal- and plant-based foods [26,27] showing a high probability to be horizontally transferred into human pathogens [28].The results obtained were subjected to statistical analysis, including cluster analysis and principal coordinates analysis (PCoA).

## 2. Materials and Methods

### 2.1. Preparation of H. Illucens Rearing Substrates

Nine different substrates were prepared for the rearing of *H. illucens* larvae. Cs obtained from a mixture of Robusta and Arabica varieties of roasting coffee (Saccaria caffè S.R.L., Marina di Montemarciano, AN, Italy) was used as a basal rearing substrate (control). The Cs was frozen at −20 °C and then ground to a 2 ± 0.4 mm particle size before the preparation of rearing substrates as detailed by Truzzi et al. [9,24]. The experimental substrates were prepared by including increasing percentages (5%, 10%, 20%, and 25%) of freeze-dried *S. limacinum* (S) or *I. galbana* (I) microalgae in the basal Cs substrate. The organically produced microalgae were provided by AlghItaly Società Agricola S.R.L. (Sommacampagna (VR), Italy). No antibiotics were used for the production of microalgae. An aliquot of each of the rearing substrates was stored at −20 °C until further analysis.

### 2.2. Rearing of H. Illucens Larvae

Six-day-old larvae of *H. illucens* purchased from Smart Bugs s.s. (Ponzano Veneto, TV, Italy) were reared on 9 different substrates at the experimental facility of the Department of Agricultural, Food and Environmental Sciences (Polytechnic University of Marche, Ancona, Italy). The *H. illucens* were divided into 9 groups, each containing 750 larvae, and then further divided into 5 replicates, each containing 150 larvae. The feeding rate within the plastic boxes was 100 mg day^−1^ until the prepupae stage was reached (after approximately 1 month). The larvae were transferred to new substrates once a week during the rearing period. No antibiotics were used for the commercial and experimental rearing of *H. illucens* larvae. The details of the rearing conditions are as reported by Truzzi et al. [9,24]. At the end of the experiment, larvae, and frass from each of five replicates per substrate type were collected manually under sterile conditions, pooled together to reduce the effect of biological variation, and stored at −20 °C until analysis.

### 2.3. DNA Extraction

The rearing substrates, *H. illucens* larvae and their frass (10 g each) were added to 90 mL of sterile physiological solution (0.85% NaCl, *w v*^−1^) and homogenized in a Stomacher machine (400 Circulator, International PBI, Milan, Italy) for 5 min at 260 rpm. Aliquots (1.5 mL) of the obtained homogenates (dilution 10^−1^) were centrifuged for 10 min at 14,000 rpm to collect the cell pellets, which were further used for DNA extraction using an E.Z.N.A. soil DNA extraction kit (Omega Biotek, Norcross, GA, USA). The quantity and purity of the extracted DNAs were determined by a Nanodrop ND 1000 (Thermo Fisher Scientific, Wilmington, DE, USA) and standardized to a concentration of 25 ng μL^−1^ before further analysis. Moreover, the effective extraction of the bacterial DNA was checked by PCR amplification of the bacterial 16S rRNA gene using the universal prokaryotic primer pair 27F-1495R [29].

### 2.4. Detection of AR Genes by PCR and Nested PCR

The extracted DNAs were screened by PCR for the detection of 12 AR genes conferring resistance to erythromycin [*erm*(A), *erm*(B), *erm*(C)], tetracycline [*tet*(K), *tet*(M), *tet*(O), *tet*(S)], vancomycin (*vanA*, *vanB*), β-lactams (*blaZ, mecA*) and aminoglycosides (*aac-aph*). To increase the detection sensitivity, the negative PCR amplification products for each AR gene in the study were further subjected to a nested PCR assay. The primers used for the PCR and nested PCR as well as the thermal cycling conditions for all but the *aac-aph* gene were as previously reported by Milanović et al. [30], while the PCR conditions for the latter gene were detailed by Garofalo et al. [31]. DNA was extracted from 12 bacterial reference strains [16,17,19,20], each carrying one of the AR genes under study, and they were used as positive controls in each PCR and nested PCR assay, whereas the DNA extracted from *Enterococcus faecalis* JH2-2 [32] was used as a negative control. Furthermore, a blank control containing molecular biology grade water instead of DNA was used in each assay to exclude the presence of PCR contaminants. The presence/absence of AR genes in the samples was checked by analyzing the PCR and nested PCR amplification products by electrophoresis on a 1.5% (*w*/*v*) agarose gel. The correct size of the amplification products for each AR gene in the study was determined by comparison with a 100 bp DNA molecular weight marker (HyperLadder^TM^ 100 bp, Bioline, London, UK) as shown in Supplementary Figure S1. To check the specificity of PCR/nested PCR assay, the randomly selected amplification products for each AR gene in study were sent to Genewiz (Leipzig, Germany) for the purification and sequencing. The obtained sequences were subjected to Basic Local Alignment Search Tool (BLAST) analysis available from National Center for Biotechnology Information showing high similarity (≥98%) with the corresponding sequences deposited in GenBank database, thus confirming the specificity of the PCR/ nested PCR primers used in study.

### 2.5. Statistical Analysis

The detection frequency of AR genes in each sample was calculated as the total number of detected AR genes divided by the total number of tested AR genes. Similarly, the detection frequency for each AR gene in the study among the samples was calculated as the total number of positive samples divided by the number of samples.

A data matrix was created with PCR- and nested PCR-positive and PCR-negative samples scored as 1 or 0, respectively. A Jaccard similarity index matrix was adopted for hierarchical cluster analysis by applying the unweighted pair group method with the arithmetic mean (UPGMA). PCoA was carried out using NTSYS software (Applied Biostatistics Inc., New York, USA). The combination of principal coordinates allowing the best separation of the groups found by cluster analysis was identified through the bidimensional graphical representations of the PCoA scores of all samples. Following the results of the cluster analysis and PCoA, the difference in AR gene frequencies (presence vs. absence) between the categories of samples was evaluated by Pearson’s chi square test (χ^2^) (JMP statistical software version 11.0.0, SAS Inst. Inc., Cary, NC, USA). Comparisons among groups included an overall χ^2^ test followed by orthogonal contrasts.

## 3. Results

The results of the PCR and nested PCR screening of 12 AR genes conferring resistance to erythromycin, tetracycline, vancomycin, β-lactams, and aminoglycosides in nine experimental rearing substrates as well as in the *H. illucens* larvae and the resulting frass are reported in Table 1. The screening was first performed by PCR and, in the case of negative results, by a nested PCR assay. Generally, the genes conferring resistance to tetracyclines, namely, *tet*(S), *tet*(M) and *tet*(K), were detected with the highest frequencies, at 92.6%, 88.9%, and 63.0% of the samples, respectively, followed by the erythromycin resistance gene *erm*(B), detected in 59.3% of the samples. In contrast, the *blaZ* and *vanB* genes were never identified. Nested PCR allowed the detection of most of the analyzed AR genes with the *erm*(A), *mecA*, and *aac-aph* genes detected exclusively by this method. Generally, the frass samples were characterized by the largest number of different AR genes.

Considering rearing substrates, the *tet*(M) and *tet*(S) genes were widely present in all analyzed substrates, with the *tet*(K) gene found in Cs as well as in substrates containing *I. galbana* and in substrate CsS25 prepared by the highest inclusion level of *S. limacinum* (25%). Regarding erythromycin resistance genes, *erm*(B) was detected sporadically among different substrates. The *aac-aph* gene was detected only in the substrates containing 10% of both microalgae (CsS10 and CsI10). Finally, a larger number of AR genes was detected in substrates prepared with *I. galbana* than in those containing *S. limacinum*, irrespective of the inclusion percentages.

Regarding *H. illucens* larvae reared on experimental substrates, no positive results were obtained after the first round of PCR for the larvae grown on substrates containing *S. limacinum*, while the larvae HCsI20 and HCsI25 reared on substrates containing *I. galbana* were positive for the *erm*(B) and *tet*(M) genes after the first PCR. Moreover, the absence of *erm*(A) and *aac-aph* as well as the *tet*(K) gene was observed in the larvae reared on substrates prepared with *S. limacinum* and *I. galbana*, respectively. The genes detected with the highest frequencies were *tet*(S), *tet*(M), *tet*(O), and *erm*(B), whereas the *erm*(A) and *aac-aph* genes were found only in *H. illucens* larvae reared on substrates containing high percentages (>20%) of *I. galbana.* The highest number (50%) of AR genes was detected in *H. illucens* larvae grown on the substrate containing 25% *I. galbana* (HCsI25).

In contrast to the samples of rearing substrates and *H. illucens* larvae, where the AR genes were mostly detected by nested PCR, most of the frass samples were positive even after the first round of the PCR assay for the *erm*(B), *erm*(C), *tet*(M), and *tet*(S) genes. The *mecA* and *erm*(C) genes were found exclusively in frass samples collected after the rearing of larvae on substrates supplemented with *I. galbana*, except for FCsS20, which also resulted in positivity for the *erm*(C) gene.

**Figure 1:**
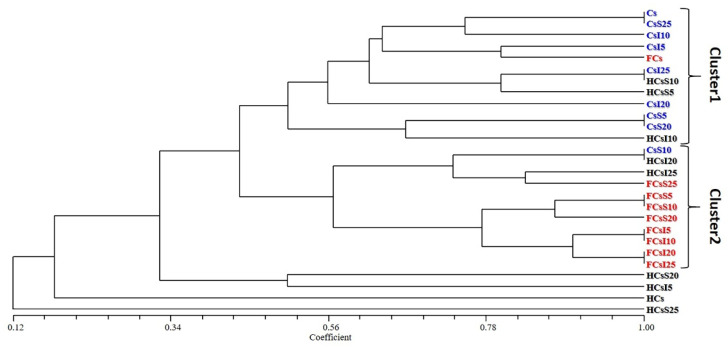
Hierarchical clustering dendrogram plotted from the cluster analysis. Cs, rearing substrate composed of 100% coffee silverskin; CsS5, CsS10, CsS20, CsS25 and CsI5, CsI10, CsI20, CsI25, rearing substrates supplemented with 5%, 10%, 20%, and 25% *Schizochytrium limacinum* and *Isochrysis galbana*, respectively; HCsS5, HCsS10, HCsS20, HCsS25 and HCsI5, HCsI10, HCsI20, HCsI25, *Hermetia illucens* larvae reared on the corresponding substrates; FCsS5, FCsS10, FCsS20, FCsS25 and FCsI5, FCsI10, FCsI20, FCsI25, frass collected from the *H. illucens* larvae reared on the corresponding substrates. Color-coding: feeding substrates (blue), *H. illucens* larvae (black), and frass (red).

Cluster analysis (Figure 1) identified two main clusters and four samples of *H. illucens* larvae that showed a low similarity with the two clusters and among each other. The results of the cluster analysis are discussed based on the grouping of three main categories of samples that were investigated for the occurrence of AR genes, namely, feeding substrates, larvae and frass.

The majority of the substrate samples were grouped in Cluster 1, with the exception of CsS10 being grouped in Cluster 2. All frass samples inoculated with both *S. limacinum* and *I. galbana* (FCsI and FCsS) were included in Cluster 2, whereas the FC sample (control) was grouped in Cluster 1. Moreover, several frass samples in Cluster 2 shared the same AR gene profile (FCsS5 and FCsS10, FCsI5 and FCsI10, FCsI20, and FCsI25).

The larval samples were included in both clusters, with HCsS and HCsI mainly grouped in clusters 1 and 2, respectively, whereas HCsS25 was clearly different from the other analyzed samples. Therefore, the *H. illucens* samples were characterized by a higher variability in AR gene distribution than the feeding substrates and frass samples.

**Figure 2:**
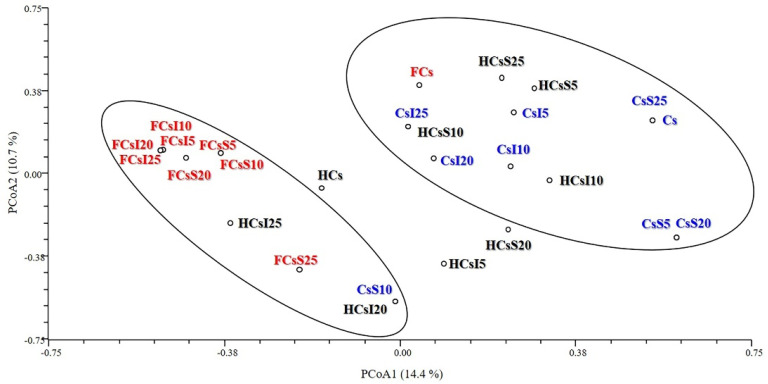
Principal coordinate analysis (PCoA) plot: principal coordinates 1 and 2. Cs, rearing substrate composed of 100% coffee silverskin; CsS5, CsS10, CsS20, CsS25 and CsI5, CsI10, CsI20, CsI25, rearing substrates supplemented with 5%, 10%, 20%, and 25% *Schizochytrium limacinum* and *Isochrysis galbana*, respectively; HCsS5, HCsS10, HCsS20, HCsS25 and HCsI5, HCsI10, HCsI20, HCsI25, *Hermetia illucens* larvae reared on the corresponding substrates; FCsS5, FCsS10, FCsS20, FCsS25 and FCsI5, FCsI10, FCsI20, FCsI25, frass collected from the *H. illucens* larvae reared on the corresponding substrates. Color-coding: feeding substrates (blue), *H. illucens* larvae (black), and frass (red).

**Figure 3:**
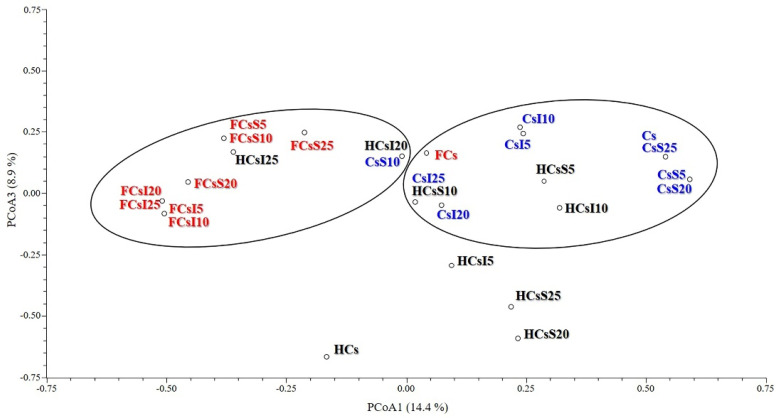
Principal coordinate analysis (PCoA) plot: principal coordinates 1 and 3. Cs, rearing substrate composed of 100% coffee silverskin; CsS5, CsS10, CsS20, CsS25 and CsI5, CsI10, CsI20, CsI25, rearing substrates supplemented with 5%, 10%, 20%, and 25% *Schizochytrium limacinum* and *Isochrysis galbana*, respectively; HCsS5, HCsS10, HCsS20, HCsS25 and HCsI5, HCsI10, HCsI20, HCsI25, *Hermetia illucens* larvae reared on the corresponding substrates; FCsS5, FCsS10, FCsS20, FCsS25 and FCsI5, FCsI10, FCsI20, FCsI25, frass collected from the *H. illucens* larvae reared on the corresponding substrates. Color-coding: feeding substrates (blue), *H. illucens* larvae (black), and frass (red).

PCoA (Figure 2 and Figure 3) confirmed the differentiation between Clusters 1 and 2. In particular, the first two principal coordinates explained 25.12% of the total variation and separated Cluster 1 from Cluster 2 (Figure 2). The third principal coordinate contributed to 8.86% of the total variance and moved the four isolated samples away from the two main clusters (Figure 3).

Due to the results of the cluster analysis and PCoA, the AR gene frequencies detected in the substrates, larvae, and frass were calculated and compared by the χ^2^ test. As shown in Table 2A, the absolute frequencies of the AR genes highlighted clear differences among the groups of samples, confirmed by the highly significant overall χ^2^ test (Table 2B). Orthogonal contrasts showed a significant difference between frass and the other two groups, as shown by the first contrast. No differences were found between the rearing substrates and the larval groups (contrast 2, Table 2B). In general, genes coding for resistance were prevalent in the frass samples (65%) compared to the rearing substrate (30%) and the larval (29%) samples.

## 4. Discussion

The DNAs extracted from the pooled samples of *H. illucens* larvae, their rearing substrates and corresponding frass samples were screened for the occurrence of 12 AR genes using PCR assays. The samples that were negative for each of the AR genes under study were further subjected to nested PCR assays designed to enhance the gene detection sensitivity, as previously reported by Garofalo et al. [31]. The high sensitivity of the latter method was confirmed in recent studies aimed at detecting the same AR genes in different edible insects [14,16,17,19,20] as well as in human stool and saliva samples [30,33].

In the present study, nested PCR allowed the detection of the higher number of the analyzed AR genes with respect to the first round PCR. The *erm*(A), *mecA*, and *aac-aph* genes were exclusively found by nested PCR, which could be related to a small number of their gene copies in the samples, not detectable by first round PCR.

The most prevalent genes among all tested samples were those conferring resistance to tetracyclines. This is not surprising since the amount of tetracyclines used worldwide exceeds almost every other class of antibiotics, mainly due to their extensive range of activity and low cost [34]. It is noteworthy that bacterial resistance to tetracyclines has increased in the last thirty years, with 46 different genes conferring resistance to this class of antibiotics identified among various Gram-positive and Gram-negative bacteria isolated from humans, animals, and the environment [35].

Of note, to guarantee the safety of food or feed, both the ingredients and the final products should be free from microbiological contaminants, including antibiotic-resistant bacteria [36]. Regarding edible insects, during rearing, bacteria in the insect feed come into direct contact with the bacteria from the insect’s gut, thus making it possible for them to exchange AR genes [22]. In the present study, the tetracycline resistance genes *tet*(M) and *tet*(S), both encoding ribosomal protection proteins, largely prevailed among the rearing substrates. A high incidence of *tet*(M) was expected since this gene has the widest host range among all *tet* genes, principally due to its association with mobile genetic elements, which increase its transferability among bacteria [35]. Similarly, the *tet*(S) gene, showing 79% amino identity with *tet*(M), has been identified among Firmicutes and Gammaproteobacteria from a broad range of ecological sources [37]. The inclusion of *I. galbana* in the basal substrate (Cs), especially at high percentages (20 and 25%), seems to be associated with a higher prevalence of the tested AR genes when compared with substrates supplemented with *S. limacinum.* Furthermore, some of the tested genes, such as *mecA* and *tet*(O), while not found in Cs, emerged exclusively in substrates enriched with *I. galbana*, whereas the *tet*(K) gene, previously detected in Cs, largely prevailed in mixed substrates containing the latter microalgae. This finding could be due to the beneficial effect of *I. galbana* on the proliferation of bacteria carrying these genes.

To the author’s knowledge, no studies have been performed on the presence of antibiotic residues or on the occurrence of AR genes in coffee silverskin or microalgae-based substrates, whereas a similar study investigating the incidence of AR genes in organic wheatmeal used for the rearing of edible mealworms reported the exclusive presence of tetracycline *tet*(S) and *tet*(K) genes [20]. A very recent study focused on the microbial dynamics of the same rearing substrates analyzed in the present study reported the massive presence of lactic acid bacteria (LAB) belonging to the *Lactobacillus*, *Leuconostoc*, and *Weissella* genera [38]. In the study of Osimani et al. [38], the prevalence of *Leuconostoc* in the basal Cs substrate and substrates supplemented with *S. limacinum* was reported; moreover, the prevalence of *Weissella* in substrates supplemented with *I. galbana* was observed [38]. Although LAB have been recognized as safe with a QPS (qualified presumption of safety) status given by the EFSA [39], the frequent detection of LAB carrying AR genes that can be horizontally transferred to pathogens through the food chain is of great concern [40].

Despite the growing interest of the scientific community in *H. illucens* larvae for bioconversion and bioremediation, limited information is available on safety aspects regarding its gut microbiota [41]. Among the limited number of studies investigating the composition of the *H. illucens* gut microbiota, only a few have focused on the effect of diet on its composition and they have shown that the gut microbiota is strongly influenced by the diet, but a stable core microbiota is also present [38,41,42,43]. Another important safety aspect neglected in most of the studies performed on *H. illucens* larvae is the occurrence of antibiotic-resistant bacteria and their AR genes, which in turn could be transferred to fish or livestock destined for human consumption.

In the present study, *H. illucens* larvae reared on the basal Cs substrate resulted positive only for the *erm*(B) and *tet*(O) genes, which, together with the *tet*(M) and *tet*(S) genes, prevailed among *H. illucens* larvae irrespective of the microalgae used for the enrichment of the substrates. Recently, Cifuentes et al. [22] detected the *tet*(M) gene in both the larval and pupal gut of *H. illucens* reared on chicken feed. Another recent study on the microbial dynamics and the occurrence of AR genes in *H. illucens* larvae fed soy meal supplemented with oxytetracycline reported the presence of 107 AR genes conferring resistance to aminoglycosides, vancomycin, macrolide-lincosamide-streptogramin B, tetracyclines, chloramphenicol, and sulfonamides, showing a notable prevalence of tetracycline resistance genes [10]. The genes conferring resistance to tetracycline, including *tet*(M) and *tet*(O), were also stably present in the gut of *H. illucens* larvae during bioconversion of manure collected from chickens treated with subtherapeutic doses of tetracyclines [21].

Although these ubiquitous genes could be associated with the *H. illucens* core microbiota, the higher number of *tet*(M) and *erm*(B) genes detected even in the first round of PCR, as well as the exclusive presence of the *erm*(A) and *aac-aph* genes in the larvae reared on the substrates enriched with *I. galbana*, could indicate a positive correlation between this microalga and the bacteria carrying these genes. The *erm*(A) gene is the most common AR determinant in staphylococci, mostly detected in methicillin-resistant strains [44], whereas the *aac-aph* gene conferring resistance to aminoglycosides has frequently been detected in Gram-positive bacteria mainly belonging to the genera *Enterococcus*, *Staphylococcus*, and *Streptococcus* and rarely in Gram-negative bacteria [45]. Finally, the highest number (6/12) of all tested AR genes was detected in larvae reared on substrates containing 25% *I. galbana* (HCsI25).

Since a strong correlation between the *H. illucens* gut microbiome and resistome has been reported in a study performed by Liu et al. [10], similar associations between the AR genes detected in *H. illucens* larvae in the present study and their microbiota highlighted by Osimani et al. [38] could be hypothesized. Indeed, Osimani et al. [38] recently reported the prevalence of *Paenibacillus* in larvae reared on basal Cs substrates, whereas *Morganella*, followed by *Paenibacillus*, *Lysinibacillus*, and *Enterococcus*, prevailed in *H. illucens* reared on substrates enriched with *I. galbana.* Multidrug-resistant species from the *Morganella* genus that are able to spread AR genes horizontally among the same or different species have been reported by Liu et al. [46]. In contrast, larvae fed substrates containing *S. limacinum* were characterized by a large prevalence of *Brevundimonas*, followed by *Enterococcus* and *Paenibacillus*. Of note, all of the identified genera include the Firmicutes or Proteobacteria phyla, both commonly associated with multiple resistances [47]. Furthermore, the Proteobacteria is recognized as a potential reservoir of genes conferring resistance to tetracyclines and comprises AR determinants for all important antibiotic resistance mechanisms [48]. Interestingly, the only two studies trying to elucidate the correlation between the microbiota and resistome of *H. illucens* larvae reported contradictory results. Indeed, Liu et al. [10] found a strong correlation between tetracycline resistance genes and *Enterococcus*, *Ignatzschineria*, *Bordetella*, *Providencia*, and *Proteus*, whereas the analyses performed by Cai et al. [21] resulted in a very poor correlation between the target AR genes and the relative abundance of genera present in the gut of *H. illucens* larvae.

Little is known about the microbiota of frass collected during the rearing of *H. illucens* [22,38,41,49], and only two studies investigated the occurrence of AR genes in insect frass collected during the rearing of *H. illucens* [22] and *T. molitor* [20] larvae. In the present study, a high contamination level of frass by bacteria carrying the AR genes under study was assumed. Similarly, an accumulation of AR determinants in feed residues collected at the end of the *H. illucens* rearing process was reported by Cifuentes et al. [22]. In the present study, the accumulation phenomenon was more evident in frass collected from larvae fed substrates enriched with microalgae, thus explaining the results of the cluster analysis and orthogonal contrasts. Furthermore, *I. galbana* could be associated with the almost exclusive presence of *erm*(C) and *mecA* genes in frass, both typically detected in staphylococci. The *erm*(C) gene is commonly responsible for erythromycin resistance in methicillin-susceptible strains [44], whereas *mecA* confers resistance against methicillin, nafcillin, oxacillin, and cephalosporins [50]. Similarly, Cifuentes et al. [22] reported the high abundance of the latter gene in feed residues without being previously detected in *H. illucens* gut samples. The same authors reported a strong correlation of the *mecA* gene with the relative abundance of staphylococci. Such a correlation could only be partly assumed in the present study since the metataxonomic analysis performed by Osimani et al. [38] revealed extremely low relative abundances for this group of microorganisms among the same frass samples. Indeed, *Brevundimonas*, followed by *Alcaligenes* and *Enterococcus*, prevailed in the frass from *H. illucens* fed *I. galbana*, whereas the predominance of *Brevundimonas*, followed by *Trichococcus* and *Myroides*, was observed in the frass from larvae fed *S. limacinum*-enriched diets. As discussed above, the identified genera belong to the Firmicutes, Proteobacteria or Bacteroides phyla, all commonly associated with multiple resistances [47,51]. The accumulation of AR genes in frass during the rearing of *H. illucens* larvae is of great concern since this waste can be used as a biofertilizer [52,53], acting beneficially on plant growth by inducing plant resistance mainly due to the presence of chitosan, a derivative of chitin [54], or more recently, as a fish diet supplement [55], thus posing potential safety risks for the environment and humans.

## 5. Conclusions

Little is still known about the occurrence of AR genes in *H. illucens*; hence, the results of the present study could represent a valuable contribution to this important safety issue, especially when *H. illucens* larvae are intended to be used as a feed or food ingredient. This study was designed to elucidate the effect of Cs-based rearing substrates supplemented with increasing percentages of *S. limacinum* or *I. galbana* microalgae on the occurrence of the selected AR genes in *H. illucens* larvae and their frass. Different inclusion percentages of microalgae did not significantly influence the distribution of AR genes in *H. illucens* larvae even if high percentages of *I. galbana* could be associated with a slightly higher prevalence of the tested AR genes. A significant accumulation of AR genes was observed in the frass, which was more evident for the samples obtained after rearing *H. illucens* larvae on substrates containing high inclusion percentages of *I. galbana*, thus raising safety concerns about reusing this waste in agriculture. The data obtained represent a first baseline to be used in future AR risk assessment in the edible insect feed chain; until then, prudent use of antibiotics during the rearing of edible insects has been recommended. Further research is needed to better clarify the correlation between the copy number of AR genes carried by the microbial communities occurring in the rearing substrates and those contained in the insect gut and in the resulting frass using quantitative PCR assays.

## Figures and Tables

**Table 1:** Antibiotic resistance (AR) genes detected by PCR and nested PCR.

Sample	AR Genes												% of Positivity for AR Genes
	*erm*(A)	*erm*(B)	*erm*(C)	*vanA*	*vanB*	*tet*(M)	*tet*(O)	*tet*(S)	*tet*(K)	*mecA*	*blaZ*	*aac-aph*	
Cs	-	-	-	-	-	■	-	■	■	-	-	-	25.0
CsS5	-	-	-	-	-	■	-	■	-	-	-	-	16.7
CsS10	-	■	-	-	-	■	-	■	-	-	-	■	33.3
CsS20	-	-	-	-	-	■	-	■	-	-	-	-	16.7
CsS25	-	-	-	-	-	■	-	■	■	-	-	-	25.0
CsI5	■	-	-	-	-	■	-	■	■	-	-	-	33.3
CsI10	-	-	-	-	-	■	-	■	■	-	-	■	33.3
CsI20	-	■	-	-	-	■	-	■	■	■	-	-	41.6
CsI25	-	■	-	-	-	■	■	■	■	-	-	-	41.6
% of positive substrate samples for each AR gene	11.1	33.3	0.0	0.0	0.0	100	11.1	100	66.7	11.1	0.0	22.2	
HCs	-	■	-	-	-	-	■	-	-	-	-	-	16.7
HCsS5	-	-	-	-	-	■	■	■	■	-	-	-	33.3
HCsS10	-	■	-	-	-	■	■	■	■	-	-	-	41.6
HCsS20	-	-	■	-	-	-	-	■	-	-	-	-	16.7
HCsS25	-	-	-	-	-	-	-	-	■	-	-	-	8.33
HCsI5	-	■	■	-	-	■	-	■	-	-	-	-	33.3
HCsI10	-	-	-	-	-	■	■	■	-	-	-	-	25.0
HCsI20	-	□	-	-	-	□	-	■	-	-	-	■	33.3
HCsI25	■	■	-	-	-	□	■	■	-	-	-	■	50.0
% of positive *H. illucens* samples for each AR gene	11.1	55.6	22.2	0.0	0.0	66.7	55.6	77.8	33.3	0.0	0.0	22.2	
FCs	■	-	-	-	-	□	■	□	■	-	-	-	41.6
FCsS5	■	■	-	-	-	□	■	□	■	-	-	■	58.3
FCsS10	■	□	-	-	-	□	■	□	■	-	-	■	58.3
FCsS20	■	■	□	-	-	□	■	□	■	-	-	■	66.6
FCsS25	■	□	-	-	-	□	-	□	-	-	-	■	41.6
FCsI5	■	□	□	□	-	□	■	□	■	■	-	■	83.3
FCsI10	■	□	□	□	-	□	■	□	□	■	-	■	83.3
FCsI20	■	□	□	-	-	□	■	□	□	■	-	■	75.0
FCsI25	■	□	□	-	-	□	■	□	□	■	-	■	75.0
% of positive frass samples for each AR gene	100	88.9	55.6	22.2	0.0	100	88.9	100	88.9	44.4	0.0	22.2	
Overall % of positive samples for each AR gene	40.7	59.3	25.9	7.4	0.0	88.9	51.9	92.6	63.0	18.5	0.0	44.4	

□, sample positive after PCR; ■, sample positive after PCR and nested PCR; -, negative sample; Cs, rearing substrate composed of 100% coffee silverskin; CsS5, CsS10, CsS20, CsS25 and CsI5, CsI10, CsI20, CsI25, rearing substrates supplemented with 5%, 10%, 20%, and 25% *Schizochytrium limacinum* and *Isochrysis galbana*, respectively; HCsS5, HCsS10, HCsS20, HCsS25 and HCsI5, HCsI10, HCsI20, HCsI25, *Hermetia illucens* larvae reared on the corresponding substrates; FCsS5, FCsS10, FCsS20, FCsS25 and FCsI5, FCsI10, FCsI20, FCsI25, frass collected from the *H. illucens* larvae reared on the corresponding substrates. Color-coding: feeding substrates (blue), *H. illucens* larvae (black), and frass (red).

**Table 2:** Results of the chi-square (χ^2^) test: (**A**) AR gene frequencies of each group and (**B**) total χ^2^ and orthogonal comparisons.

**(A) Antibiotic Resistance (AR)**			
Group	Presence *		Absence *
Feeding substrates (Cs)	32 (30)		76 (70)
*Hermetia illucens* larvae (H)	31 (29)		77 (71)
Frass (F)	70 (65)		38 (35)
**(B) χ^2^ Test**			
Comparisons	χ^2^	*p*-value	OR (95% CI)
Overall test (Cs vs. H vs. F)	37.83	<0.0001	
Comparisons	χ^2^	*p*-value	OR (95% CI)
Orthogonal comparisons			
1. (Cs + H) vs. F	37.81	<0.0001	4.47 (2.74–7.32)
2. Cs vs. H	0.02	n.s.	0.96 (0.53–1.72)

* the relative frequencies (%) are reported in brackets. n.s., not significant; OR, odds ratio; CI, confidence interval.

## Supplementary Materials

The following are available online at https://www.mdpi.com/2073-4425/12/2/213/s1, Figure S1: Representative gel electrophoresis images of PCR (panel A) and nested PCR (panel B) amplification products of 12 target antibiotic resistance (AR) genesscreened in the present study.
