# Supplementary material for: Occurrence of Antibiotic Resistance Genes in Hermetia illucens Larvae Fed Coffee Silverskin Enriched with Schizochytrium limacinum or Isochrysis galbana Microalgae
Source: Genes (Basel). 2021 Feb 1;12(2):213. doi: 10.3390/genes12020213 (PMC7912857; doi:10.3390/genes12020213)
Supplement: Supplementary file 1 [file genes-12-00213-s001.pdf]

**Supplementary Figure S1.** Representative gel electrophoresis images of PCR (panel A) and nested PCR (panel B) amplification products of 12 target antibiotic resistance (AR) genes screened in the present study.

A)

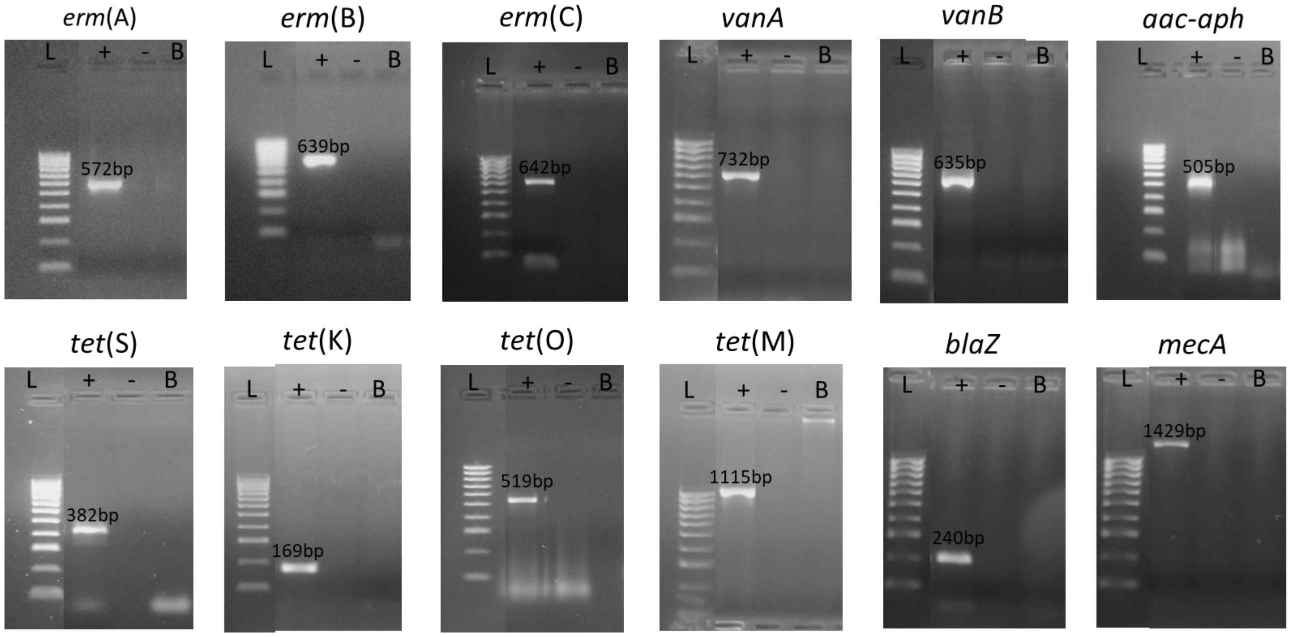


B)

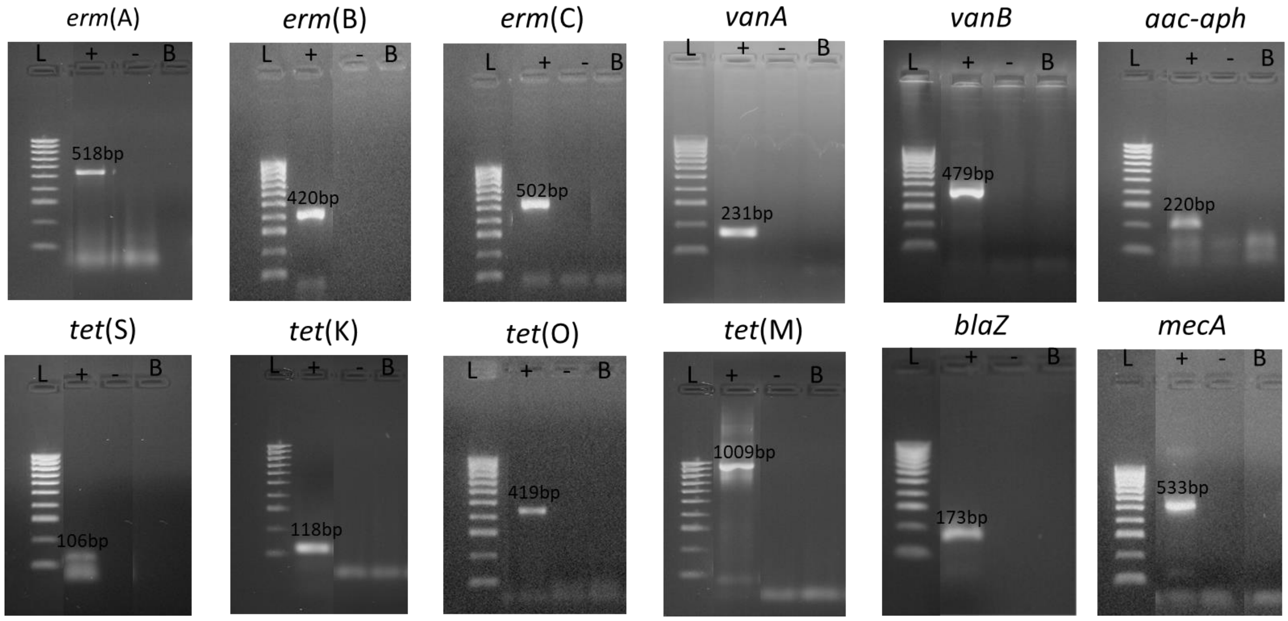


L, HyperLadderTM 100 bp (Bioline, UK); +, positive PCR control; -, negative PCR control; B, blank PCR reaction. The correct size of the amplification products for each AR gene was determined by comparison with a 100 bp DNA molecular weight marker (L).
